# Effects of Weather on Coronavirus Pandemic

**DOI:** 10.3390/ijerph17155399

**Published:** 2020-07-27

**Authors:** Qasim Bukhari, Joseph M. Massaro, Ralph B. D’Agostino, Sheraz Khan

**Affiliations:** 1 McGovern Institute for Brain Research, Massachusetts Institute of Technology (MIT), Cambridge, MA 02139, USA; skhan7@mgh.harvard.edu; 2 Department of Biostatistics, Boston University School of Public Health, Boston, MA 02215, USA; jmm@math.bu.edu; 3 Department of Mathematics and Statistics, Boston University, Boston, MA 02215, USA; ralph@bu.edu; 4 Department of Radiology, Massachusetts General Hospital, Harvard Medical School, Boston, MA 02114, USA; 5 Athinoula A. Martinos Center for Biomedical Imaging, Massachusetts General Hospital, Harvard Medical School, Massachusetts Institute of Technology, 149 13th Street, CNY-2275, Boston, MA 02129, USA

**Keywords:** coronavirus, humidity, temperature, tropical, COVID-19, COVID, weather

## Abstract

The novel coronavirus (SARS-CoV-2) has spread globally and has been declared a pandemic by the World Health Organization. While influenza virus shows seasonality, it is unknown if COVID-19 has any weather-related affect. In this work, we analyze the patterns in local weather of all the regions affected by COVID-19 globally. Our results indicate that approximately 85% of the COVID-19 reported cases until 1 May 2020, making approximately 3 million reported cases (out of approximately 29 million tests performed) have occurred in regions with temperature between 3 and 17 °C and absolute humidity between 1 and 9 g/m^3^. Similarly, hot and humid regions outside these ranges have only reported around 15% or approximately 0.5 million cases (out of approximately 7 million tests performed). This suggests that weather might be playing a role in COVID-19 spread across the world. However, this role could be limited in US and European cities (above 45 N), as mean temperature and absolute humidity levels do not reach these ranges even during the peak summer months. For hot and humid countries, most of them have already been experiencing temperatures >35 °C and absolute humidity >9 g/m^3^ since the beginning of March, and therefore the effect of weather, however little it is, has already been accounted for in the COVID-19 spread in those regions, and they must take strict social distancing measures to stop the further spread of COVID-19. Our analysis showed that the effect of weather may have only resulted in comparatively slower spread of COVID-19, but not halted it. We found that cases in warm and humid countries have consistently increased, accounting for approximately 500,000 cases in regions with absolute humidity >9 g/m^3^, therefore effective public health interventions must be implemented to stop the spread of COVID-19. This also means that ‘summer’ would not alone stop the spread of COVID-19 in any part of the world.

## 1. Introduction

In the beginning of 2020, several cases of novel coronavirus, called as 2019-nCoV or COVID-19, appeared across China [1,2]. The disease quickly spread to other regions, due to its highly transmissive nature [3] and increased global mobility, and was declared a pandemic by the World Health Organization on 11 March 2020 [4].

Human coronaviruses have been associated with a wide spectrum of respiratory diseases in different studies [5,6,7,8,9]. Influenza viruses have been observed to follow a seasonal pattern [10,11] and SARS-Cov-1, (a type of coronavirus), was also found to lose its ability to survive longer at higher temperatures [12], which may be due to the breakdown of their lipid layer at higher temperatures [13]. Some seasonality has also been observed for other coronaviruses [14] however it remains unclear how COVID-19 will vary with changes in climatic conditions, since it is the very first time that it emerged in the human population. Initial reports from China [15,16,17] found conflicting results on the relationship of weather patterns with COVID-19 cases. 

There have been stark differences in temperatures and humidity between countries that are affected worse by the COVID-19 pandemic and those that escaped a similar scale of outbreak. The temperature and humidity ranges that seem to correspond to lower reported incidence rates, are geographically found between 30 N and 30 S which are circles of latitude that are 30 degrees north and south of the Earth’s equatorial plane, respectively. The lack of large numbers of cases in the countries between 30 S and 30 N (<5,000,000 i.e., approximately 15% of total COVID-19 cases as of 1 May 2020), even though they account for more than 40% of the global population, is perplexing. It has been suggested that many of these countries are lagging behind and an exponential jump in the number of COVID-19 cases is 2 to 3 weeks behind North America and Europe. The number of COVID-19 cases in these countries could, in fact, be high and the low number of confirmed COVID-19 cases is due to a lack of reporting and testing in many of these countries such as Pakistan, Indonesia and several African nations, which have an underdeveloped health infrastructure. However, many countries and regions such as Singapore, UAE, Saudi Arabia, Australia and Qatar have performed more COVID-19 tests per million people than US, Italy, and several European countries [18], suggesting that non-testing is not an issue at least for some countries between 30 S and 30 N. India recently did hundreds of thousands of tests, yet the reported number of cases per million people did not rise significantly. The incidence rate of COVID-19 cases in all these countries are lower than American and European countries, even though most of them have not implemented drastic quarantine measures such as those in China and Europe until recently. Many of these countries, such as UAE (more than 15 million visitors annually), Singapore (more than 18 million visitors annually) and Qatar (approximately 2 million visitors annually), are also global travel hubs with thousands of people entering and exiting the country every day. Saudi Arabia was visited by hundreds of thousands of pilgrims from around the world on a regular basis until 27 February 2020. In the light of these facts, it is worth exploring other factors governing the incidence and spread of COVID-19. For each country and state, the epicenter of COVID-19 is large and well-connected cities and the role of connectivity and population in the spread of a pandemic is well established. Beyond population and connectivity, other factors including health care system, demographics, social structure, public policy and climate can govern the extent and rate of the spread of SARS-CoV-2. In this paper, we explored the association (if any) between weather and the transmission of SARS-CoV-2 as climate appears to be one of the major differences between the countries/regions experiencing high and low incidence rates.

## 2. Data and Methods

### 2.1. Weather Data

We obtained weather data using the ‘worldmet’ library in R (a programming language for statistical analysis) from 20 January 2020 to 1 May 2020. We downloaded temperature, relative and humidity and wind speed at a time step of 15 min and used the daily mean values for our analysis. We also calculated absolute humidity using Clausius Clapeyron equation [19,20] as follows

(1) AH =6.112 ∗ e^17.67TT+243.5∗ RH ∗ 2.1674273.15+ T 

where AH is the absolute humidity, RH is the relative humidity and T is the temperature in degrees °C.

For Australia, Canada and US weather data were obtained at provincial/state level and for the remaining countries, the data were obtained at country level. For each country/state, we downloaded the weather data from the busiest airport to approximate the weather of that state/country (for example, Logan Airport for Massachusetts, USA and Icheon of South Korea).

### 2.2. COVID-19 Cases

The data on confirmed Covid-19 cases for each country and state (where available) were downloaded from John Hopkins University Coronavirus Resource Center repository on 1 May 2020.

### 2.3. Relation Between COVID-19 Cases and Weather

For visualizing, we divided the entire data in six periods (period 1—20 Jan 2020 to 15 Feb 2020, period 2—16 Feb 2020 to 29 Feb 2020, period 3—1 March 2020 to 15 March 2020, period 4—16 March 2020 to 30 March 2020, period 5—1 April 2020 to 15 April 2020 and period 6—16 April 2020 to 1 May 2020). For each period, we obtained the number of new confirmed Covid-19 cases and analyzed the weather data of all the countries/states in which new cases were observed to uncover the relationship between COVID-19 outbreak and weather.

## 3. Results and Discussion

**Figure 1:**
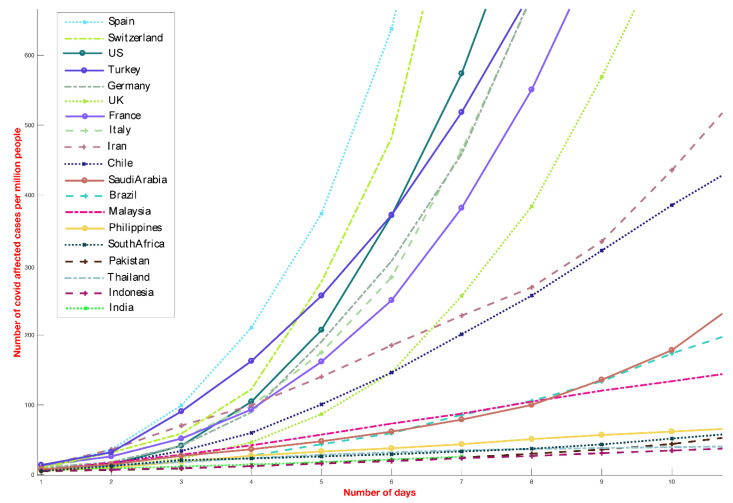
Incidence curve of COVID-19 cases for different countries (until 1 May 2020). The figure shows the total number of cases (normalized by the population of the country) starting from 5 persons affected per million. Different regions and states clearly follow different incidence curves. The *y*-axis has been capped at 600 cases per million. The countries were chosen as the top 9 countries from cold and dry weather and top 10 from hot and humid weather by 1 May. The countries are listed in Table S1, together with their classification of hot, humid/cold, dry and other demographics and weather information.

**Figure 2:**
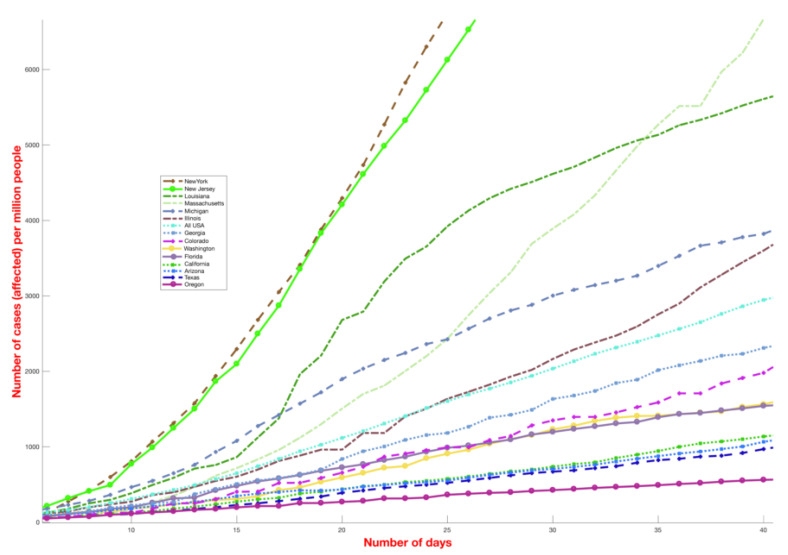
Incidence curve of COVID-19 cases for different US states (until 1 May 2020). The figure shows the total number of cases (normalized by the population of the state) starting from 20 persons affected per million. The *y*-axis has been capped at 6000 cases per million.

**Figure 3:**
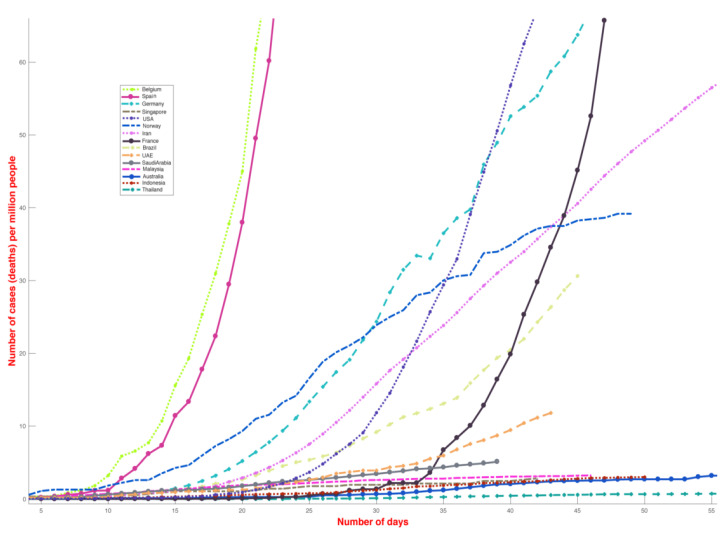
Cumulative death rate curve of COVID-19 deaths for select different countries (until 1 May 2020). The figure shows the total number of deaths (normalized by the population of the country). Different regions and states clearly follow different curves of death rate. The *y*-axis has been capped at 60 cases per 1,000,000 population.

The analysis presented in this paper provides a direct comparison between the number of COVID-19 cases globally and local environmental conditions and is motivated by the significant differences in the reported incidence COVID-19 cases among different countries (Figure 1) as well as different US states (Figure 2). Countries and states experiencing high incidence rates such as Italy, France, Germany, New York, Massachusetts and other northern US states exhibit weather patterns similar to the original hotspots of Hubei and Hunan, with mean temperatures between 3 and 10 °C in February and March (Figure 1). On the contrary, countries with warmer humid climates such as Malaysia, Thailand and other South-East Asian countries (Figure 1) exhibit a lower growth rate. This divide is even visible within the US (Figure 2). The demographic and other weather information of countries mentioned in Figure 1 is reported in Table S1, where the largest cities data are used for average dew point and temperature values. The number of COVID-19 deaths for selected different countries (until 1 May 2020) has been shown in Figure 3.

**Figure 4:**
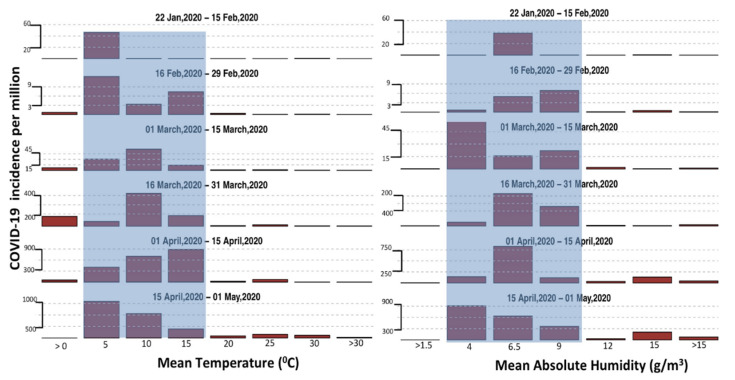
Number of COVID-19 cases across the world as a function of temperature and absolute-humidity. The figure shows the number of cases reported COVID-19 cases per 10-day period for different temperature and absolute humidity values. Temperature and absolute humidity range with the majority of COVID-19 cases (>85%) is highlighted with light blue color and is consistently between 3 and 17 °C for temperature and between 4 and 9 g/m^3^ for absolute humidity every week.

Our analysis shows that for each 10-day period between 22 January 2020 and 1 May 2020 the maximum number of new cases are reported in regions with mean temperature between 0 to 17 °C and absolute humidity between 1 and 9 g/m^3^. The data to date show that the number of cases for temperature >17 °C and absolute humidity >9 g/m^3^ is low throughout for each time period (Figure 4). After 11 March, we observe a surge in the number of cases in regions with temperature >17 °C, however it is still many times lower than the surge in cases 15 °C (Figure 4). The sudden increase in the number of cases in regions with temperature >17 °C could be due to several reasons, including recent initiation of large-scale COVID-19 testing in India, Brazil, Indonesia and Pakistan or analysis of backdated samples (Florida, US) [21,22] (discussed subsequently). However, over time, this surge in cases in the hot and humid countries died out, and the exponential growth was not sustained, despite no strict lockdown measures being put in place by the respective governments. Our initial work [23] submitted to SSRN website indicated cases until March 30 only, and we raised points that UAE and Malaysia may look more like cold dry countries and the next few weeks would make this more clear. However, now, when several weeks have passed, it is evident that the number of cases in UAE and Malaysia have not increased as rapidly as in countries with cold and dry weather. 

The number of COVID-19 cases detected in a country/state depends on multiple factors including testing, population (density), community structure, social dynamics, governmental policies, global connectivity, air and surface life [24], reproduction number and serial interval of the SARS-CoV-2. Many of the information regarding COVID-19 in still emerging, such as being airborne for more than 3 h and having different survival times on metals, cardboards and plastics [24] (published on March 17, 2020). The most recent work on the stability of SARS-CoV-2 with changes in temperature showed that the virus is sensitive to heat [25]; however, these findings are still very preliminary. Previous works have shown that the spread of viruses depends upon environmental factors, with many respiratory pathogens showing seasonality and decreased transmission rates in warmer humid climates, however, the behavior of COVID-19 is still under investigation and also the subject of this paper. Below, we analyze the possible reasons for the low number of reported COVID-19 cases to date in regions with T > 17 °C and AH > 9 g/m^3^ which includes most of the countries between 30 S and 30 N. 

First, around 81% of testing was conducted in non-tropical countries (30 N and above), and 85% of the COVID-19 cases were recorded in the same countries within a temperature range of 0 to 17 °C and absolute humidity (AH) between 1 and 9 g/m^3^. Therefore, the higher number of tests and global connectivity of the northern-cooler countries may partially explain the difference in the number of confirmed COVID-19 cases between cooler and warmer-humid regions. However, several countries and regions between 30 S and 30 N that performed extensive testing including Singapore, UAE, Saudi Arabia, Australia, Qatar etc., reported a lower number of positive COVID-19 cases per capita and a lower incidence rate than European and American countries, suggesting that the discrepancy is not solely due to lack of testing and there might be other factors might be playing a role in the spread of COVID-19.

While most tropical countries have under-tested for COVID-19, if the extent of COVID-19 cases were comparable to those in European and American cities, the mortality rates [26] would have provided some indication of the extent and severity of the spread (Figure 3). Although mortality rate depends on several factors, including the demographics, age and health of the population, the lack of good health facilities and prevalence of large numbers of cases could lead to a higher mortality rate. However, the mortality rate in South Asian countries is lower, even though the health infrastructures in many of these countries are substantially inferior to European and American infrastructures. Additionally, reports of crowded hospitals and people suffering from respiratory sickness would have emerged in local media, which have typically not been reported. In Italy, pro-active testing on COVID-19 was limited until the healthcare system was overwhelmed, with a large number of people showing up with symptoms associated with COVID-19, thus forcing the government to take adequate measures to slow the spread of COVID-19. The apparent difference between these regions has also been reported extensively [27].

It could be argued that countries between 30 S and 30 N are in the early phase of the pandemic where the incidence rate appears linear and would pick up only after hundreds of cases have been reported, a phenomena observed for several countries including the US, Italy, and Spain. It is possible that several countries will see an exponential growth soon if they are in the initial phase of the pandemic. However, for several countries such as Australia, Malaysia, Thailand and Saudi Arabia that have reported more than 1500 cases, the number of days it took to reach these levels is much slower than European countries and American states. The incidence rate in Indonesia, Vietnam and Cambodia is also low, but we do not discuss them here due to an inadequate number of testing per capita. Furthermore, in Figure 1, we compared the top nine cold and dry countries with the top 10 hot and humid countries by April 1. This gives them enough weeks to multiply their already high number of reported cases in the coming weeks. However, as the graph shows, several of these countries in hot and humid places showed a much slower incidence growth.

It could also be argued that the government in these countries is taking exceptional measures to stop the spread of the virus, which we know is not true. Until March 26, 2020, most Asian and African countries were working business as usual. India, the 2nd most populous country in the world, started a lockdown on March 26, 2020 and Indonesia (4th most populous country) did not implement any lockdown until very recently [28]. Even though the proportion of tests across all the countries between 30 S and 30 N are much lower than the countries above 30 N, the fraction of confirmed cases, that is, 500,000 positive cases out of around 7 million tests performed [29] (approximately 6 out of 100) is lower for countries between 30 S and 30 N than countries above 30 N, that performed around 29 million tests and found approximately 3 million positive COVID-19 cases [29] (approximately 10 out of 100) as of May 3, 2020. If countries between 30 S and 30 N were only testing highly symptomatic people, the proportional of confirmed cases should have been higher and not lower. Therefore, the lower number of cases to date, in the densely populated countries between 30 and 30 N (combined population of almost 3 billion people) may be due to non-human factors that might play a role in slowing the spread of the virus. Viral transmission has been suggested to be lower at higher humidity and temperature, and the majority of countries between 0 and 30 N are warm–humid. Therefore, the role of environmental factors in the spread of COVID-19 is worth exploring. Data until 1 May 2020 (Figure 4) reveal that 85% of the COVID-19 cases have been recorded in the regions within a temperature range of 0 to 17 °C and absolute humidity between 1 and 9 g/m^3^, and, similar to other coronaviruses which show seasonality [14], warm–humid conditions might be a factor contributing to the lower number of cases in tropical countries, resulting in a slower virus spread, as has been observed for other viruses.

The relation between the number of COVID-19 cases and temperature and the absolute humidity observed here is consistent for every time period included in this analysis between 22 January 2020 and 1 May 2020 (Figure 4) and is based on approximately 3.5 million confirmed cases globally, although the underlying reasoning behind this relationship is still not clear. Similarly, we do not know which of the environmental factors is more important. It could be that either temperature or absolute humidity is more important, or both may be equally or not important at all in the transmission of COVID-19. The temperature dependency of COVID-19 may be similar to that of SARS-Cov-1, which loses its ability to survive in higher temperatures [12], due to the breakdown of their lipid layer at higher temperatures [13]. The relationship between COVID-19 and temperature and absolute humidity could also be secondary, and the lower spread in the tropics might be due to longer exposure to sunlight (as it may have antiviral properties), the naturally occurring ultraviolet (UV) radiation in the sunlight, and other factors that are still unknown. There are preliminary lab-based findings that suggest the role of temperature, humidity and UV radiation; however, these need to be confirmed in repeated experiments [25,30]. It may also be argued that the immunity level of people living in those places is relatively high (since wide-spread silent tuberculosis (TB) is also common in their population) and researchers at an Australia institute have started vaccinating health workers working with COVID-19 patient with BCG (Bacillus Calmette–Guérin) vaccine [31] to test the vaccine’s effectiveness against COVID-19. However, the exact factors underlying these differences must be further explored through laboratory testing, especially the role of environmental factors in the spread of COVID-19, as several people, including renowned epidemiologists [32], have suggested a link, but none have provided definitive proof backed by rigorous laboratory testing.

With summer approaching, it may also be that the temperature ranges we have seen for most of the cases (0 to 17 °C) to date may be further broadened in the coming weeks, and any effect may only happen at higher temperature similar to that found in SARS-Cov-1 [12]. In fact, in light of the early lab test reports [25], it would be reasonable to assume that if there is any temperature-related effect, it would only happen at temperatures well above 30 °C. The humidity dependency may be due to the less effective airborne nature of the viruses at higher absolute humidity, thus reducing the overall indirect transmission of COVID-19 at higher levels of humidity [33]. Although higher humidity may increase the amount of virus deposited on surfaces, and virus survival time in droplets on surfaces [33], the reduction in the virus spread by indirect (through air) transmission may be the factor behind the reduced COVID-19 spread in humid climate. These explanations are speculative and based on patterns observed for other coronaviruses. Urgent study/experiments on the association between coronavirus transmission against temperature and humidity in laboratories are needed to understand these associations.

We opted for not using *R*_0_ (basic reproduction number) in our work, since the *R*_0_ values reported for COVID-19 differ widely from each other [34], similar to the 1918 influenza pandemic, where various models resulted in a broad range of published values [35]. For a current pandemic such as COVID-19, where we have data that are continually updated and highly dependent upon the surveillance system, we preferred not to use *R*_0_ or *R_effective_* because the results can vary significantly depending upon the estimated *R*_0_ (or *R_effective_*) [36]. In fact, few papers published to date have shown contradictory results on the effect of climatic factors on the spread of COVID-19 [36,37,38,39,40].

## 4. Conclusions

Our conclusions are based on the currently available data with several unknowns, including how the virus is mutating and evolving, case fatality ratio, reproductive numbers and direct versus indirect transmissions. The relation between temperature and humidity and the spread of COVID-19 cases should be closely monitored and studied under different climatic conditions in controlled laboratory settings. If a strong environmental dependence in the spread of COVID-19 emerges, then it should be used to optimize the COVID-19 mitigation strategies. Our results in no way suggest that COVID-19 would not spread in warm humid regions and effective public health interventions should be implemented across the world to slow down the transmission of COVID-19.

Implications for future transmission of COVID-19: In April 2020, more than 300,000 cases have been recorded in regions with T > 17 °C, even though this is much lower than the number of cases observed in regions with T between 0 and 17 °C. The rise in cases in the warmer parts of Europe including the Andalusian region and southern US states provides little hope that rising temperature alone could lower the spread of cases in the current hotspot of Europe and the US, as the mean temperature for most of the major cities in these regions is below 25 °C for most of May and June. Therefore temperature could have an effect similar to the relationship observed between SARS-CoV-1 and temperature [12]; however, it might be at much higher temperatures, as reported in the early laboratory experiments [25], and at the mild summer temperatures of Europe and North America, the virus may continue to survive for several days on plastics and metals [24]. The southern part of Florida and Louisiana are the only places in the US which have started experiencing temperatures >17 °C (65 °F) and absolute humidity above 9 g/m^3^ since the end of March. In our previous work [23], we reported these states as outliers. However, once these temperature and humidity ranges arrived in those places, the increase in cases significantly dropped, as evident from the decrease in cases after April 10, 2020. However, it must be mentioned that the weather alone may not have been responsible for these decreased numbers, rather, the lockdown measures and precautions may have had a larger impact.

In comparison to temperature, absolute humidity is tightly bound throughout (Figure 4) with 85% of cases occurring between 1 and 9 g/m^3^. It has been suggested that absolute humidity plays an important role in the transmission of viruses [33,41,42] and its role in the transmission of COVID-19 should be thoroughly investigated. We calculated the theoretical absolute humidity for temperatures between −5 and 40 °C and RH between 0 and 100% (see Equation (1)). The absolute humidity is <9 g/m^3^ for temperature below 15 °C (Figure 4). For temperatures between 15 and 25 °C, the relative humidity has to be higher than 60% for absolute humidity to be greater than 9 g/m^3^. Based on currently available data, COVID-19 is spreading easily in regions with absolute humidity <9 g/m^3^. This, once again, has serious implications for the assumption that the COVID-19 spread would slow down during summer in the current hotspots of US and Europe, as, in many regions, the absolute humidity might be above 9 g/m^3^ (Figure 4) only for a brief period in July and August. On the other hand, Asian, African and South American countries, especially those experiencing high absolute humidity levels >9 g/m^3^ during summer, have already started experiencing those high ranges of temperatures and humidity, and therefore, the rise in number of cases there has been significantly lower than in cold and dry countries. Furthermore, most of these countries also have large populations, and even a small percent of the COVID-19 positives could lead to many hundreds of thousands of cases in absolute numbers. Therefore, these hot and humid countries may not get any further relief during summer, and must rely on better healthcare precautionary measures to limit the spread of COVID-19. Moreover, it is still not clear what other factors are important, as monsoon in many of these hot and humid regions is known to increase the transmission of influenza, especially in lower middle class people who live in tight spaces and are near to each other during the rainy season. Further, if solar radiation is also an important factor (typically lower in monsoon due to cloud cover) then the spread of COVID-19 might be confounded by these opposing factors. Therefore, while the effect with temperature and humidity has recently been also confirmed through laboratory testing [25], it is important to note that any effect that weather has may be limited compared to other factors, including but not limited to social distancing, quarantine measures, and cultural practices.

Recent spike in COVID-19 cases in warmer and humid regions: Since mid-March, several countries in Asia, South America and Africa started showing an increase in the number of COVID-19 cases. It is suggested that many countries will show an exponential increase in the number of COVID-19 cases. However, many of these countries were not testing for COVID-19 cases until the beginning of March, and therefore a spike was expected once they start testing. We should also account for the difference between local transmission and exported cases (where people got the infection elsewhere but were tested in another region). Unlike the stringent lockdown imposed by the European and the US authorities, movement within and outside the country in the Middle East, Africa, South America and South Asia remained reasonably fluid until mid-March. Most of the initial cases in Pakistan [43] and Qatar [44] were imported from Iran [43,44], and Pakistan has not implemented proper lockdown measures to date. In fact, a partial lock that was implemented in Pakistan nullifies the effect of any social distancing. In Australia, the majority of the cases were also imported, and in the last few weeks the number of cases in Australia dropped significantly. India did not start testing until very recently either, so there was a spike in cases. However, as several weeks have passed since then, and we now have data to compare against, we can safely say that the incidence rates in these countries are not close to that in New York, Spain or Italy. Furthermore, several of these countries have performed hundreds of thousands of tests, specially Indian subcontinent (India, Pakistan and Bangladesh) together accounting for 22% of global population, with India already performing approximately 1 million tests (as of 1 May), and Pakistan performing more than 150,000 tests, and found fewer COVID-19 positive cases than the cold and dry countries. Similarly, Saudi Arabia, has also performed more than 300,000 tests, however, they found fewer positive cases than many of the cold and dry countries.

Brazil, Louisiana and Florida seemed to be an outlier as the number of COVID-19 cases rose rapidly in mid-March, even though the number of cases per capita in Brazil and Florida remained much lower than northern regions. The high number of cases in Louisiana might be due to the Mardi Gras festival [45], where thousands of people gathered from across the world in February. Late testing can also explain the initial jump in cases in Brazil and Florida in early March. However, once the government took precautionary actions and implemented quarantine measures, and temperature and humidity levels grew higher in mid-March, the increase in cases slowed down, especially since mid-April. Compared to New York, and New Jersey, where the cases continue to rise exponentially despite strict lockdown measures, the difference is quite striking. The recent rise in cases in other South American countries with hot and humid weather, including Peru and Ecuador, may be due to the lack of early testing and late response to implementing social distancing measures. This also stands as an example that warm and humid regions will continue to see an increase in cases until proper measures are implemented.

Limitations: There may be several caveats to our work. Even though the number of reported cases was taken directly from the WHO reports, several countries are underreporting cases and have adopted different public health strategies [46]. For example, while Korea did widespread testing to identify potential COVID-19-positive subjects, including asymptomatic ones, to reduce transmission, the US and several other countries, including the UK and Japan, decided to only test individuals with symptoms or in contact with COVID-19-positives. This may, in turn, lead to undetected cases, and therefore, even though the population may have a larger number of COVID-19-positives, they may go undetected until it is transmitted to the most vulnerable in the population. Additional climatic factors such as solar radiation and cloud cover were not considered in our analysis, which could have also played an important role in the spread of COVID-19; however, since solar radiation and cloud cover are correlated with temperature in natural environments, similar patterns can be expected from those climate variables. Further, the rate of outdoor transmission versus indoor and direct versus indirect transmission are also not well understood, and environmental-related impacts are mostly applicable to outdoor transmissions. Recently there has been an increase in cases in warm and humid places, which may be due to other factors including but not limited to social distancing, quarantine measures and cultural practices.

Recommendations: Several governments have adopted the ‘flatten the curve’ strategy to mitigate the burden on healthcare instantly, spreading the number of patients requiring treatment over time to properly manage the medical needs of the patients, and eventually slowing the spread of the pandemic in summer. While proper quarantine measures help in ‘spreading the curve’, we believe that warm humid conditions in the coming days would be unlikely to be of any use in most of Europe and North America, if climate plays a role in the spread of COVID-19.

We highly stress the importance of using the proper quarantine measures, even in warmer, humid regions where the incidence rate appears to be lower, to effectively reduce the transmission of COVID-19 and protect the vulnerable against it. Warm weather alone may not be able to stop the spread of COVID-19, even in hot and humid countries, if proper safety measures are not taken, since we have evidence already that COVID-19-positive cases also keep increasing in hot and humid regions. Besides weather, there are several other factors that may play roles in the number of affected cases in any region, including population density, public health policies, political and social structures, healthcare quality, healthcare intervention, and global connectedness. Future work should include these factors, and use an epidemiological statistical model to further investigate the relationship between weather and COVID-19 transmission.

## Supplementary Materials

The following are available online at https://www.mdpi.com/1660-4601/17/15/5399/s1, Table S1: Basic demographic and weather data of the countries from Figure 1.
