# Supplementary material for: Effects of Weather on Coronavirus Pandemic
Source: Int J Environ Res Public Health. 2020 Jul 27;17(15):5399. doi: 10.3390/ijerph17155399 (PMC7432279; doi:10.3390/ijerph17155399)
Supplement: Supplementary file 1 [file ijerph-17-05399-s001.pdf]

Table S1: Basic demographic and weather data of the countries from Figure 1

Countries	Category	Location	Population	Average Temp in February	Average Temp in March	Average Temp in April	Average dew point in February	Average dew point in March	Average dew point in April
		(in latitude, longitude)	(in million)	(in C)	(in C)	(in C)	(in C)	(in C)	(in C)
USA	*Cold and Dry*	37.09° N, 95.71° W	327	1.8	5.7	11.5	-6.2	-5.7	-2.6
Italy	*Cold and Dry*	41.87° N, 12.56° E	60.48	3	6.8	10.7	-1	2	5
Spain	*Cold and Dry*	40.46° N, 3.74° W	46.66	7.2	9.8	11.7	1	2	4
Germany	*Cold and Dry*	51.16° N, 10.45° E	83.02	-1	4	7	-3	-2	1
France	*Cold and Dry*	46.22° N, 2.21° E	67	5.6	8.8	11.5	2	3	3
Iran	*Cold and Dry*	32.42° N, 53.68° E	81.16	5	10	17	-3	-2	-1
UK	*Cold and Dry*	55.37° N, 3.43° W	66.65	4.5	6.9	8.7	1	2	3
Switzerland	*Cold and Dry*	46.81° N, 8.22° E	8.57	1.3	5.3	8.8	-2	2	2
Turkey	*Cold and Dry*	38.96° N, 35.24° E	82	5	7	12	1	2	6
Brazil	*Hot and Humid*	14.23° S, 51.92° W	209.3	27.5	26.6	24.7	21	21	20
Chile	*Hot and Humid*	35.67° S, 71.54° W	18.73	20	18	15	11	10	8
Malaysia	*Hot and Humid*	4.21° N, 101.97° E	31.62	27	27	28	73	74	75
Philippines	*Hot and Humid*	12.87° N, 121.77° E	106..7	26	27	29	70	70	72
Pakistan	*Hot and Humid*	30.37° N, 69.34° E	212.2	21	25	28	8	14	19
India	*Hot and Humid*	20.59° N, 78.96° E	1353	17	22.5	28.8	46	50	52
Thailand	*Hot and Humid*	20.59° N, 78.96° E	69.04	28	29	30	20	23	24
Saudi Arabia	*Hot and Humid*	23.88° N, 45.07° E	32.94	16	21	26	1	2	2
Indonesia	*Hot and Humid*	0.78° S, 113.92° E	264	26	27	27	23	23	23
South Africa	*Hot and Humid*	30.55° S, 22.93° E	57.78	21	20	17	15	14	12
